# Predicting medication adherence using ensemble learning and deep learning models with large scale healthcare data

**DOI:** 10.1038/s41598-021-98387-w

**Published:** 2021-09-23

**Authors:** Yingqi Gu, Akshay Zalkikar, Mingming Liu, Lara Kelly, Amy Hall, Kieran Daly, Tomas Ward

**Affiliations:** 1 The Insight Centre for Data Analytics, Dublin City University, Dublin 9, Ireland; 2 HealthBeacon Ltd, Dublin, Ireland

**Keywords:** Electrical and electronic engineering, Scientific data, Computational science

## Abstract

Clinical studies from WHO have demonstrated that only 50–70% of patients adhere properly to prescribed drug therapy. Such adherence failure can impact therapeutic efficacy for the patients in question and compromises data quality around the population-level efficacy of the drug for the indications targeted. In this study, we applied various ensemble learning and deep learning models to predict medication adherence among patients. Our contribution to this endeavour involves targeting the problem of adherence prediction for a particularly challenging class of patients who self-administer injectable medication at home. Our prediction pipeline, based on event history, comprises a connected sharps bin which aims to help patients better manage their condition and improve outcomes. In other words, the efficiency of interventions can be significantly improved by prioritizing the patients who are most likely to be non-adherent. The collected data comprising a rich event feature set may be exploited for the purposes of predicting the status of the next adherence state for individual patients. This paper reports on how this concept can be realized through an investigation using a wide range of ensemble learning and deep learning models on a real-world dataset collected from such a system. The dataset investigated comprises 342,174 historic injection disposal records collected over the course of more than 5 years. A comprehensive comparison of different models is given in this paper. Moreover, we demonstrate that the selected best performer, long short-term memory (LSTM), generalizes well by deploying it in a true future testing dataset. The proposed end-to-end pipeline is capable of predicting patient failure in adhering to their therapeutic regimen with 77.35 % accuracy (Specificity: 78.28 %, Sensitivity: 76.42%, Precision: 77.87%,F1 score: 0.7714, ROC AUC: 0.8390).

## Introduction

Failure to comply with the proper use of medication is a major concern globally. The World Health Organization has claimed that up to 50% of medications prescribed for long term conditions are not taken as intended. We refer to this challenge as ‘the medication adherence problem’. In this context, ‘medication’ refers to prescribed medicine(s) and ‘adherence’ refers to the extent to which the person’s behaviour corresponds with the agreed recommendations from a health care provider^1^. Shortcomings in patients’ medication adherence has a direct impact on therapeutic efficacy with concomitant increases in risk of harm to the patients themselves and the creation of significant inefficiencies and unnecessary costs to the many stakeholders involved^2^. For instance, patients who do not adhere to their heart failure (HF) medication regimens are 1.95 times more likely to die of HF than those who are adherent to medication, and the risk for coronary heart disease (CHD) for the patients who stop taking their beta-blockers is 4.5 times greater than that of patients who persist with therapy^3,4^. In contrast, persistent adherence can significantly improve clinical outcomes and can result in decreased consumption of medical resources and costs^3^. In one particular study, adherence to asthma medications by elderly patients yielded a 20% decrease in hospitalization rates^5^. Another compelling example concerns patients with schizophrenia where hospitalization was reduced by 40% for those patients who were adherent to medication^6^. These and other studies have well established the fact that improved adherence to medication therapy can significantly improve clinical and economic outcomes especially for patients on a long-term regimen^7^.

Nonadherence is a multifactorial problem that can be influenced by a range of patient-, disease-, condition-, social/economic- and healthcare system-related factors^8^. It is common for patients to be confused by treatment schedules, forget to take their medications due to unexpected events, discontinue to take their medication due to side effects, or stop taking the treatment because they feel they no longer need it. Patients that self-inject medication at home offer a particular challenge in terms of medication adherence. Currently, many medications for illnesses such as arthritis, diabetes, severe asthma, Crohn’s disease, psoriasis, growth disorder and multiple sclerosis are self-administered by injection at the patient’s home, away from a supervised healthcare provider’s environment. Medication adherence reflects a patient behaviour in following the prescribed recommendations of health care providers. Identifying those patients who tend towards poor adherence behaviour can reduce risk of hospitalization and adverse health outcomes, creating impact both at the level of the individual, where a failure in adherence leads to poor clinical outcomes, and at the societal level, where inappropriately taken medication impacts on the quality of data available for drug discovery and enhancement activities. While the state of the art comprises several approaches for predicting patients overall adherence behaviour, in this work we are focused on the prediction of adherence behaviour at the scale of individual medication adherence opportunities as captured through a medication event monitoring system. The ability to make predictions of adherence at this time scale not only allows new opportunities for improving patient behaviour towards better adherence rates, but also helps therapeutic service providers decide when to introduce following-up interventions which can help address the non-adherence problem. One particular use case is that fine grained adherence prediction allows interventions to be delivered in the correct context. In other words, the ability to predict patient behaviour at the next medication event allows personalised interventions as appropriate, for example the correction of deteriorating adherence in the case that non-adherence is predicted or supportive, reinforcing messaging in the case of likely adherence at the next therapeutic opportunity. To the best of our knowledge, adherence prediction for patients who take medication in a home setting has not been previously investigated in the literature using machine learning approaches. Specifically, our research question in this work is to determine to what degree we can predict adherence at the individual patient level using drop history data available from the medication event monitoring system. Thus, the key differences between our paper and the state of the art are that: (1) we demonstrate prediction of adherence for patients who self-administer injections in their own home; (2) our proposed model primarily relies on historic patient injection (drop) data only, collected through an internet of things (IoT)-connected smart sharps bin (SSB). Historic drop data has been demonstrated to be a more important predictor variable compared to patient-related factors such as age, gender and habits^9^; (3) the dataset used contains data from approximately 8,000 SSB units and 342,174 historic injection records, much larger that any similar dataset described in the literature to date. This paper describes data mining and predictive analytics innovations, with an emphasis on the use of injection drop data to predict the tendency of individual patients to fail to adhere to their prescribed self-injection at the next scheduled treatment time. When the group who are most likely to be non-adherent can be accurately identified and targeted, the efficiency of interventions can be significantly increased^10,11^. This work then is a step towards realising such a goal.

The remainder of the paper is structured as follows: Section “Related work and background” provides more detailed background to the problems of medication adherence measurement and prediction, with emphasis on the SSB developed both for data collection and helping patients to remember to take their medication in a timely manner. Section “Methodology” describes the methods used to build the end-to-end data analytics pipeline. Section “Model performance comparisons” illustrates the results of model performance tests and gives a comprehensive comparison between different approaches. Section “Discussion” is a discussion of the results while Section “Conclusion” concludes the paper through highlighting the significance, benefits and impact of the work from a deployment perspective.

## Related work and background

The use of machine learning has become increasingly commonplace in digital health despite the regulatory challenges which have been introduced for medical device manufacturers. The compelling performance of using machine learning in extracting useful information from large-scale healthcare data both for tracking and prediction means its adoption can disrupt and transform healthcare industries and lead to market-changing innovations. In this paper, we focus on the use of two approaches from machine learning, namely ensemble learning and deep learning. In the following, we present a brief review on both approaches in the context of adherence prediction.

### Ensemble learning for adherence prediction

Ensemble Learning is a subfield of machine learning which has been proven to be successful and scalable in a wide variety of real-world applications. Ensemble learning aims to blend various learning algorithms into a unique model that is more powerful than any individual algorithm. As different models generally do not make all of the same errors on the testing set^12–14^ a natural consequence of such an ensemble is that performance improves. As for classical machine learning, a large body of work has been found in the use of machine learning techniques to predict patient adherence. For instance, Karanasiou et al.^15^ applied eleven classification algorithms such as support vector machine (SVM) and Bayesian Networks to predict the adherence of patients with heart failure based on a dataset of 90 patients, and Franklin et al.^16^ aimed to predict patient adherence in the next 30, 60 and 90 days using ten different machine learning models based on information from Medicare enrolment files and medical and pharmacy claims.

### Deep learning for adherence prediction

Deep learning is also a subfield of machine learning. Instead of making predictions using multiple models as with ensemble learning, a deep learning architecture involves a deeply layered neural networks which can crudely mimick the behaviour of human brain in terms of computation. With neural networks, complex, non-linear relationships between inputs and outputs of a system can be effectively modelled providing sufficient data is available. Such models often exhibit better performance than traditional, classical machine learning models . As a result, deep neural networks (DNNs) have been widely used for many real-world applications in the medical and healthcare sector. For instance, DNNs have been recently applied for solving medication classification challenges in order to distinguish between patients taking either anticonvulsant and those taking no medications from electroencephalographical (EEG) data^17^. Another recent example shows that DNNs can also be used for detecting COVID-19 pneumonia^18^ from CT scans. Indeed DNNs have even been used as part of the vaccine design process for combating COVID-19 transmission^19^. Some key enabler DNNs including multilayer perceptron (MLP), convolutional neural networks (CNNs), recurrent neural networks (RNNs), long short-term memory (LSTM) have paved a new way for us to reveal deeper insight from data. Specifically, an MLP is a feed-forward neural network and can be seen as a subset of DNNs consisting of three layers, namely an input layer, a hidden layer and an output layer. It is usually seen as a fully connected network as each neuron in one layer is connected to all neurons in the next layer with activation functions applied to the input. The weights and biases associated with the neurons can be trained using backpropagation, which is an essential and effective technique to iteratively refine trainable variables, i.e. weights and biases, to best fit a model to data. CNNs are another popular and widely used approach in deep learning^20^, especially for applications in the areas of image recognition and speech processing. CNNs are based on a shared-weight architecture which utilizes filters (or kernels) that slide along input features to generate feature maps as intermediate outputs. CNNs can be seen as regularized versions of MLP. In order to deal with sequential data using neural networks, RNNs are the most popular approach to date. The key feature of an RNN is that it allows previous outputs to be used as inputs to the network, and thus it provides an effective way to capture temporal relationships between consecutive data inputs. However, RNNs have exposed several disadvantages such as vanishing/exploding gradient problems during the backpropagation through time (BPTT) process. To solve this problem, different modified RNN structures have been proposed, including LSTM among others. The key idea behind LSTM is that it leverages a memory cell as a candidate for the hidden layer output and utilize three gate control units, namely the input gate, the forget gate and the output gate. In particular, the input gate and the forget gate are used to determine to what extent the candidate memory cell output and the previous time-step memory cell output should be used for constructing the current memory cell. Finally, instead of producing the updated memory cell as the final hidden layer output, LSTM uses an output gate to control how much information is generated from the updated memory cell as the final output of the hidden layer. LSTM has shown its effectiveness in solving practical challenges such as the vanishing gradient problem seen in the simple RNN architecture. For further detailed discussions on different variants of DNNs, please see papers^20–22^.

In our previous paper, we attempted to predict patients’ adherence to self-administered injectable medication by leveraging a collection of ensemble learning models, and discussed the end-to-end pipeline we developed^23^. The new method we use here is deep learning, a subset of machine learning algorithms which has made major advances in recent years. In the literature, deep learning methods have been widely used in the field of health informatics, including, but not limited to, translational bioinformatics, medical imaging, pervasive sensing, medical informatics, and public health. A comprehensive up-to date review of research applying deep learning in health informatics associated with a critical analysis of the relative merits, and challenges of the techniques involved as well as the future outlook for the field has been reported in^24^. In particular, the use of deep learning solutions such as recurrent neural network (RNN) and long short-term memory (LSTM) for adherence forecasting has become increasingly common in recent published work^25–27^. In particular, the authors in^28^ adopted multilayer perceptrons (MLP) and convolutional neural networks (CNN) approaches to predict the adherence of type 2 diabetics using the continuous glucose monitoring (CGM) signal data collected from 9 patients. In these studies, the sample sizes are much smaller than the one considered in our paper, nevertheless these studies are still significant in establishing the feasibility and potential effectiveness of machine learning for solving such problems. In this paper, our goal is to illustrate at a much more compelling data scale the usefulness and possibilities of such technologies in understanding and potentially resolving adherence.

### Smart Sharps Bin

We employ an Internet-connected “smart sharps bin” (SSB) to track adherence with injection protocols. Designed and manufactured by HealthBeacon Ltd., the SSB is ideal for use in a home environment and records the disposal of used hypodermic needles digitally, monitors injection disposal data passively and uploads it to a long-term storage and further analysis cloud-based database to allow the support team to control drop disposals and provide follow-up services as required. This unique SSB has FDA approval and has been incorporated in North America and Europe into patient care systems^23^.

**Figure 1:**
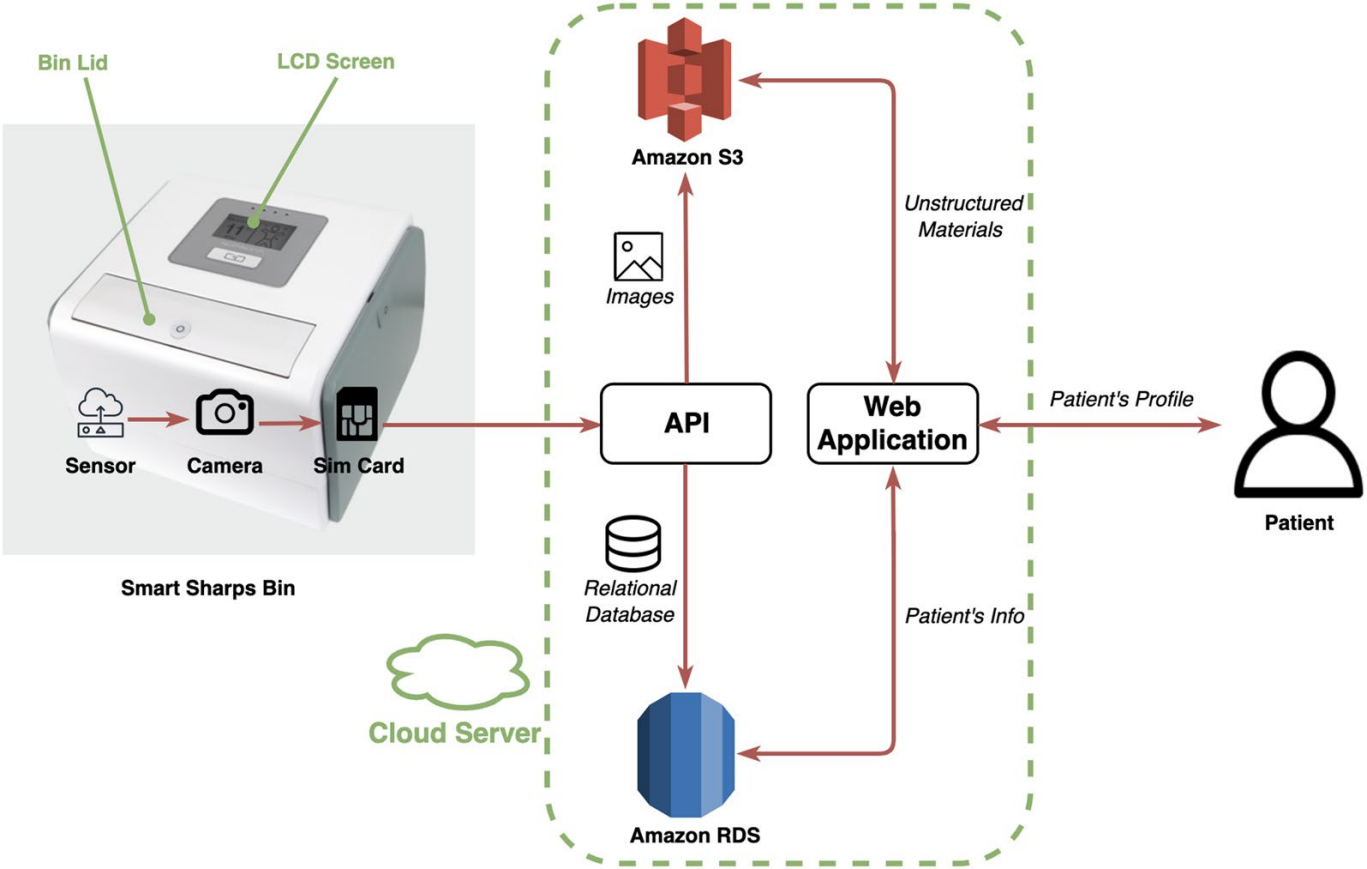
A schematic diagram of the Smart Sharps Bin.

The SSB has been developed as a home-friendly product. It consists of five parts and it is designed to require minimal involvement from the patient. The uppermost portion of the device has an LCD panel and a sharps lid embedded in it. When a patient begins a treatment program by injection, an SSB is dispatched to the patient, and preprogrammed with the patient’s personal medication schedule details, including start date, injection frequency, appropriate injection location and preferred time slots for receiving the short message service (SMS). When an injection is needed, a blue light is illuminated to draw attention to the screen of the SSB as a visual reminder to the patient. In addition, the patient is also reminded by SMS of their chosen time for the home injection. If the patient does not take their treatment, i.e. by dropping the used injector into the SSB, a SMS will be sent to the patient the following day. The patient is injected based on the prescription that is displayed on the LCD screen. After the injection is done, the patient disposes of the used injector by discarding it through the bin lid, which sits inside the SSB. When this is done, a time-stamped record is created automatically, through interruption of the sensor beam and recording by the micro-camera embedded in the SSB. Specifically, two files are saved for every drop action. One file is a comma-separated values file containing time stamp information while the second is an image file for the camera output. Both files are uploaded to the cloud server via a protected private APN and SIM Card. We note that all data are fully encrypted during this process for protection of patients’ privacy. The collected files are used for model training purposes through a dedicated data pipeline and a web application. The system architecture for interactions between the SSB and the cloud-based datastore is illustrated in Fig. 1^23^.

## Methodology

### Data acquisition and description

**Figure 2:**
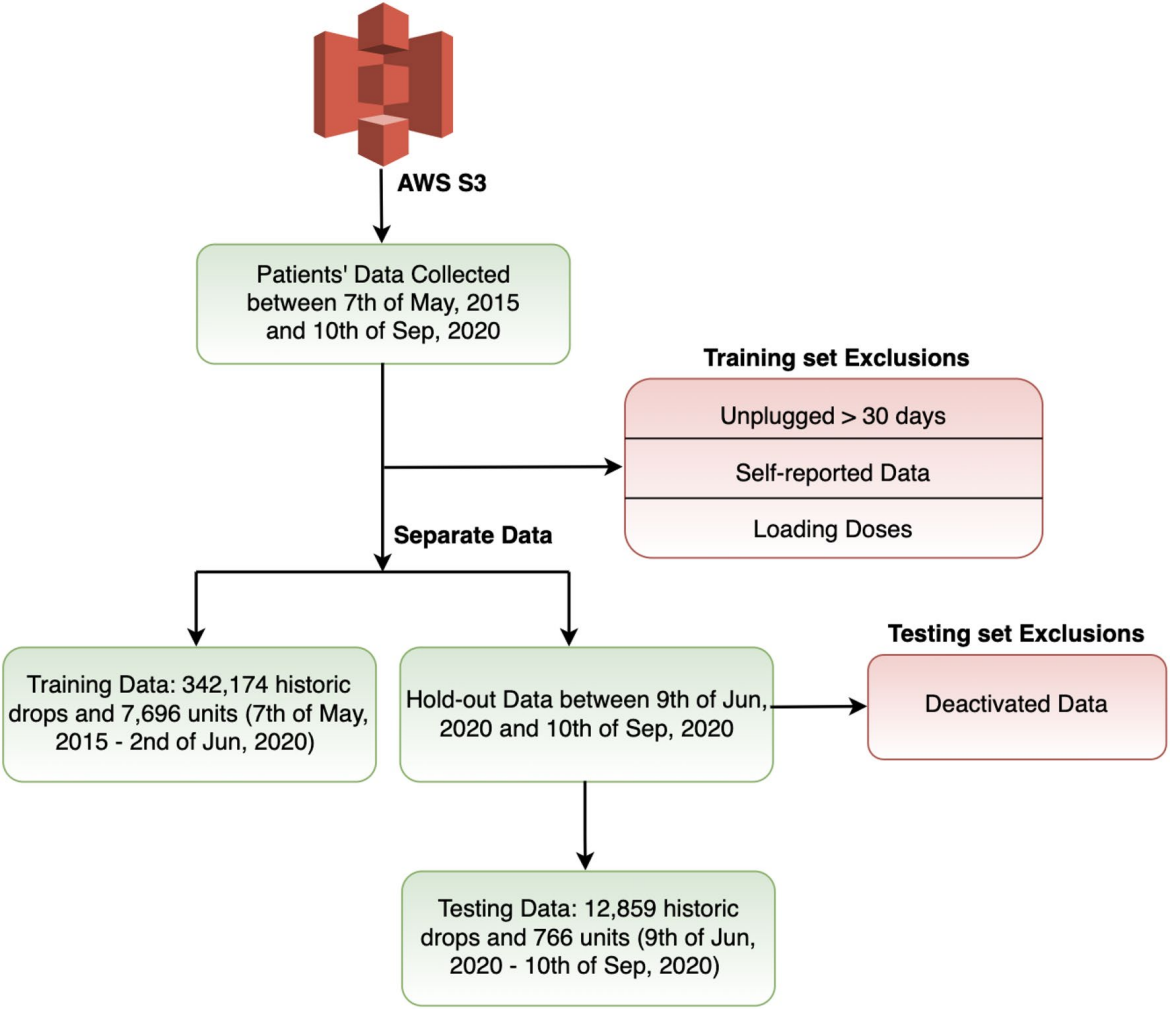
Data acquisition and division pipeline.

The dataset we used in this paper was retrieved from the cloud storage location and then further processing was conducted. Among all the extracted data, some exclusions were applied for the experiments here. For the training set, the patients’ data was removed if their units are unplugged for a period of more than 30 days before they are expected to take medications. Please note that in principle the SSB is required to remain plugged-in to support constant monitoring. However, we consider it as unplugged when the unit is not communicating for a period of more than 30 days. Also, based on self-reported data provided independently by the patients, data from the SSB was removed when patients were away from their home and the device. For the same reason, the loading doses, which are initial higher doses of medication or a series of such doses given in order to rapidly achieve a therapeutic concentration in the body, were excluded as this would introduce a bias in predicting future drops. Furthermore, if the patient’s unit is deactivated after he/she disposes drops in the SSB, such data will also be excluded when testing as there is insufficient knowledge regarding the ground truth of the patient’s drop. The data acquisition and division pipeline used in this study is shown (Fig. 2).

### Pipeline of the proposed system implementation

**Figure 3:**
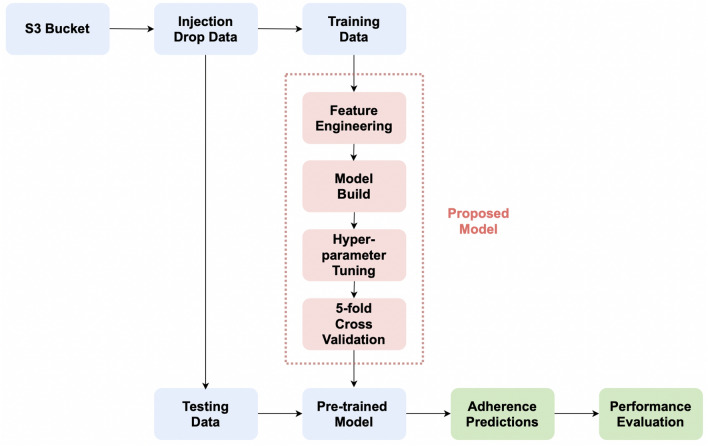
The flow chart of the proposed system implementation.

The flow chart of the proposed system implementation is shown in Fig. 3. Specifically, following the data acquisition step, the labelled patients’ data (subject to exclusions as described earlier) forms a dataset to be used for model training and evaluation. The training dataset is used to obtain the pre-trained ensemble learning/deep learning models by carrying out four specific steps including feature engineering, model building, hyper-parameter tuning and 5 fold cross validation. The detailed architecture configurations of the proposed models are discussed in “Model setting”. Next, the held-out testing data are used to estimate the adherence behaviours at the next scheduled medication date and validate the performance of pre-trained models. Finally, several performance metrics are selected for the evaluation of all learned models, the details of which are presented in “Model performance comparisons”.

### Data preprocessing

**Figure 4:**
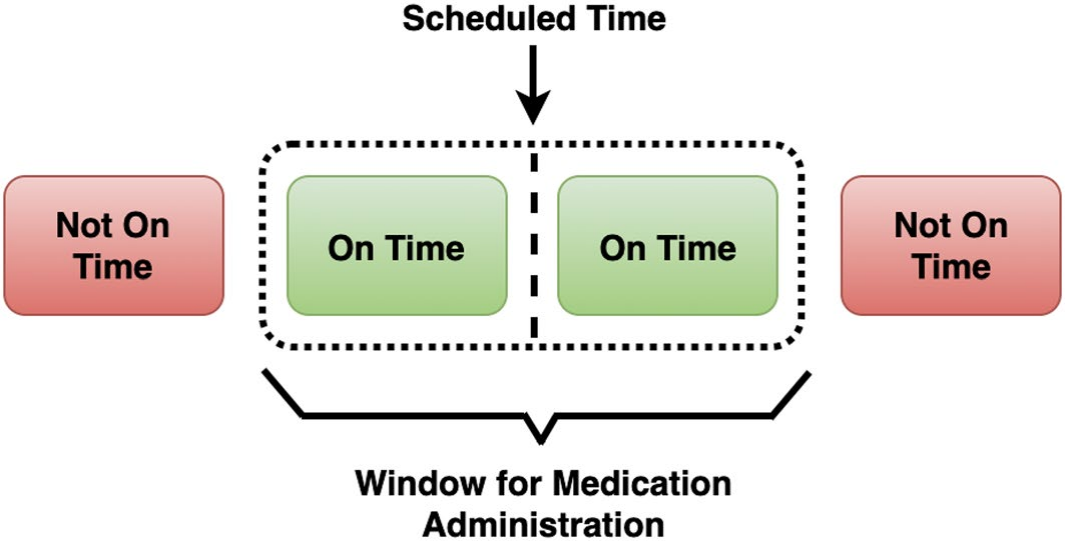
The time frame defined for “On-Time” and “Not-On-time” disposals.

The proposed models were trained and evaluated using data extracted from the SSBs. The dataset used for training the predictive machine learning models was collected from the 7th May 2015 to the 1st June 2020, inclusive. The dataset contains information obtained from 7697 HealthBeacon units, including 342,174 individual drop events. The raw dataset extracted from the cloud-based database has 130 data fields, including anonymised information regarding patients’ treatment regime, from the scheduled medication date and frequency for each patient to the drop status of scheduled injections. In order to comply with the EU General Data Protection Regulation (GDPR)^29^, data fields which contain patients’ personal data were removed from the dataset. Domain experts then recommended around 50 data fields to be evaluated for inclusion in the prediction model; fields which are believed to have a close relationship with patients’ treatment medication adherence. The dataset was then cleaned to remove empty rows/columns and redundant variables. Categorical data other than drop status was transformed into a machine learning-compatible format using one hot encoding. A new feature “day of week” was constructed based on the “time of scheduled medication event” data field. The feature “drop status” was been converted to the labels “On-Time”/“Not On-time” based on the time difference between the drop time stamp and the scheduled medication time stamp. We define the recommended time period for medication according to the prescription as “Window for Medication Administration (WMA)—in this paper. WMA varies for different drugs and different scheduled medication frequency. For instance, if the recommended time period for one drug type is 144 h, which means all drops made 72 h before or after the scheduled medication time point can be labelled as “On-Time” , within the 144 h, the behaviour can be labelled as “On-Time”, otherwise, the drop is regarded as “Not On-Time” . In the context of this work, Fig. 4 shows the drop was labelled as “On-Time” if the medication was taken within the window allowed for each patient’s SSB. The drop was marked as “Not On-time” if the medication was taken outside the WMA or was not dropped in the SSB at all.

The testing data recorded by the SSB and uploaded to the cloud database in real time for storage on a daily basis. The data collected between 9th of June, 2020 to 10th of September, 2020 with 12,859 samples are used for testing after the same pre-processing steps were applied as described above. The testing data associated with these patients’ last 6 drops was used for making adherence predictions. The testing set included the ground truth of these patients’ real adherence behaviours, and the predictions from the proposed models were compared with the ground truth adherence behaviours.

### Feature analysis and selection

In order to reduce the dimensionality of the feature vectors, selecting those most important for the prediction step, we used the Waikato Environment for Knowledge Analysis (WEKA)^30^. WEKA was used to help identify and then remove certain highly correlated features, and instead select those that contain the best predictive information, using the information gain-based feature selection technique with the built-in “InfoGainAttributeEval” attribute evaluator. The “InfoGainAttributeEval’ attribute evaluator is to calculate the information gain, also known as entropy, for each feature in the dataset in the context of the target variable (the adherence class). This technique is used to navigate different combinations of features in the dataset in order to produce a shortlist of features most relevant to the machine learning task. Here feature importance indicates the relative contribution of features in making accurate predictions on the target variable. After applying the aforementioned WEKA tools to the entire training dataset, the features selected as most relevant can be broadly classified as historic drops, medication frequency, country, and day-of-the-week for the scheduled medication event. Within this set of useful features, patient drop history emerged as the most significant feature according to this analysis. Thus, historic drop data was taken as a very significant attribute with which to make predictions of medication adherence. In order to decide the number of historic drops to take into account to form this feature-set, we incrementally increased the number of historic drops from 5 to 14 and attempted to predict the status of the next drop. Moreover, we calculated the AUC score as the performance metric, varying the number of drops from 5 to 14 evaluated using a Random Forest algorithm. The AUC score is largest when six historic drops are taken into account. Thus, we chose to use the last 6 historic drops in order to predict patient adherence for the next scheduled medication event. Consequently, the SSB units with less than 7 historic drops attached to their record were also removed from the dataset.

**Figure 5:**
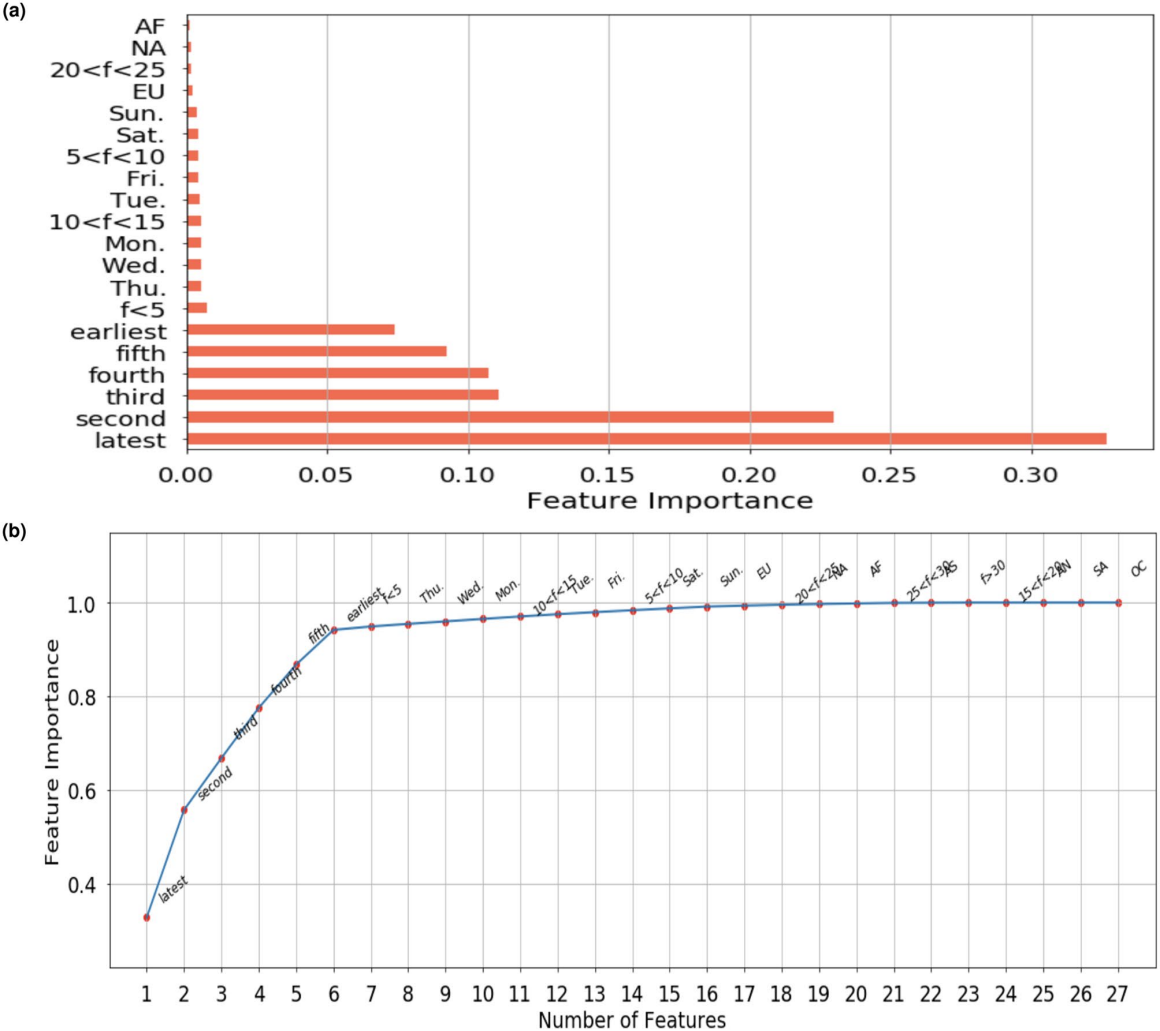
Illustration of feature importance. **(a)** The top 20 most significant features were selected by using the Extra Trees Classifier. Please note that “latest drop” refers to the most recent drop from a patient while “earliest” stands for the very first drop made in the sequence of 6 historic drops. **(b)** The accumulated importance of all selected features. The abbreviations used in the figure are summarized as follows. *f* frequency, *Mon* Monday, *Tue* Tuesday, *Wed* Wednesday, *Thu* Thursday, *Fri* Friday, *Sat* Saturday, *Sun* Sunday, *EU* European Union, *NA* North America, *AS* Asia, *AF* Africa, *AN* Antarctica, *SA* South America, *OC* Oceania.

Next, the Extra Trees Classier algorithm was used to further refine the feature selection process. Feature engineering within the training process in tree-based algorithms/models is often referred to as an embedded method^31^ and it not only offers good average predictive performance especially in highly imbalanced and high dimensional dataset^32^ but can also provide us with what we call feature importance as a way to select features. Consequently a short list of the 20 most important features were produced and these are illustrated in (Fig. 5). As before these include four main categories of features, including historic drops, day of week, medication frequency and continental location. The accumulated importance of features demonstrated the historic drops are the dominant features, constituting 94% of the feature importance, meaning that historic drop data contains the most useful predictive information, while the second most important feature is ‘day of week’ and ‘medication frequency’. Please note that here the ‘medication frequency’ refers to the frequency at which patient is scheduled to administer their injection on a regular basis, for example, once a week or daily. ‘day of week’ is the day of the week that the patient is expected to take their medication. Finally, we selected 27 of the most significant features belonging to the four categories as inputs for model training.

### Model setting

We formulated the adherence prediction problem as a binary classification problem. Considering that the number of samples in the “On-Time” class in our training set greatly exceeded those in the “Not On-Time” class, majority under-sampling was conducted for the training set. In the experiments, we chose the following metrics to evaluate model performance: accuracy, specificity, sensitivity, precision, F1 score and AUC. The ensemble learning models (XGBoost, Extra Trees, RF, GBM) and one deep learning model MLP were developed using scikit-learn, xgboost and ensemble packages in Python 3.7^33^. Some important hyper-parameters such as number of trees, maximum depth and minimum number of samples required to split an internal node were tuned for the tree-based models. The hyper-parameter tuning was done by adopting a Bayesian Optimization technique^34^ with 5-fold cross validation, which takes less computing time and has better generalisation ability on the testing set. Once appropriate hyper-parameters were selected and the models were retrained on the entire training set via 5 fold cross validation, then the mean of the selected metrics of models were reported and the pre-trained models set aside to be evaluated on the real-world future testing set.

The RNN model and LSTM model were built using Tensorflow^35^, which is capable of utilizing the parallel-processing capabilities of graphical processing units (GPUs) for fast training of deep learning models. The RNN was implemented with 3 hidden layers, and the activation function selected was the rectified linear unit (ReLU). Dropout was applied with at a rate of 0.25. We used the Adam optimizer as our optimization algorithm which is computationally efficient and has shown superior performance in many deep learning tasks^36^. The loss function used was binary cross-entropy loss^37^ as is appropriate for classification problems. Grid search was adopted for hyper-parameter tuning in this case. The model was trained with 10 epochs and a batch size of 256. The LSTM model has 3 hidden layers and uses the ReLU activation function. The model is optimized using binary cross-entropy loss and the Adam optimizer, one dropout layer was applied between the hidden layers with a rate of 0.4. The LSTM model was trained using 8 epochs and the batch size was selected as 256 in this case to gain the best result. The performance metrics figures in this paper were plotted using the Matplotlib plotting library in Python^38^.

## Model performance comparisons

In this study, we use the opportunity of this large dataset to investigate the relative performance of a number of ensemble learning and deep learning models for this task of adherence prediction, i.e. predicting if individual patients will take the self-administrative injection at the next scheduled medication opportunity. Formulating the problem this way yields a binary classification task in this case. The 7 selected models, namely, extreme gradient boosting (XGBoost), extremely randomized trees (Extra Trees), random forest (RF), gradient tree boosting (GBM), multi-layer perception (MLP), recurrent neural networks (RNN) and long short term memory networks (LSTM) were trained and validated via 5-fold cross validation. Here the cross validation technique is used to minimize bias in performance comparison and assessment. Thus, the values demonstrated in the figures are the mean values for the selected performance metrics.

**Figure 6:**
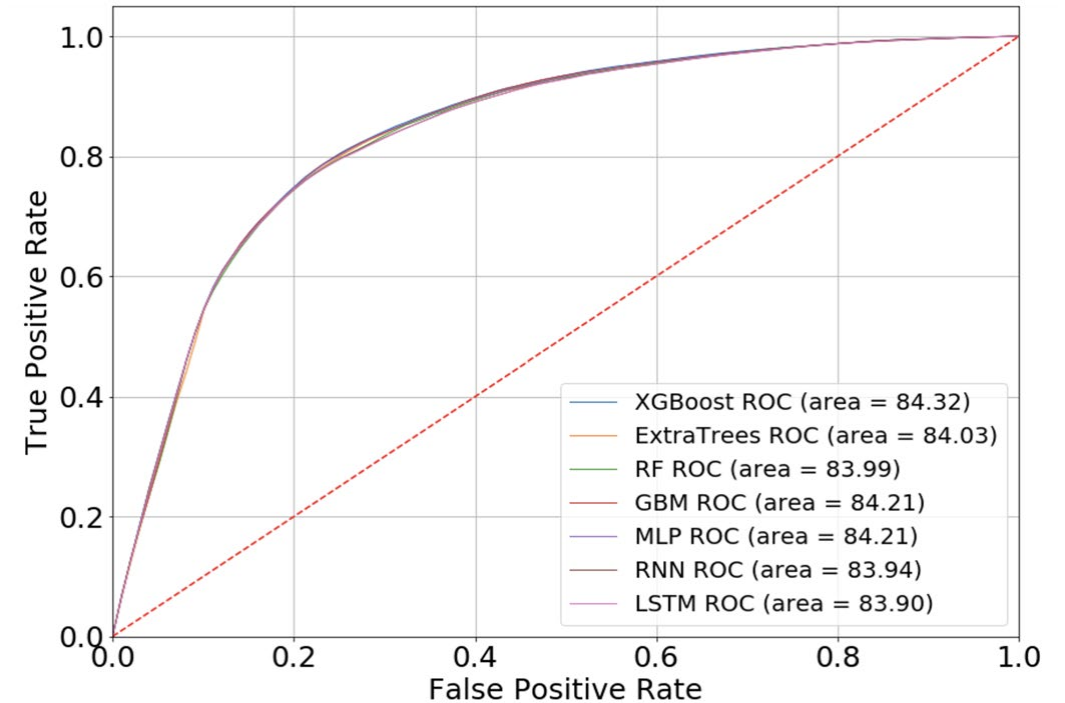
ROC curves for all selected models. The AUC scores are the average of AUCs through 5 fold cross validation. The red line represents the 50% chance. The ensemble learning classifiers include XGBoost, Extra Trees, RF, GBM. The deep learning models include MLP, RNN and LSTM.

**Figure 7:**
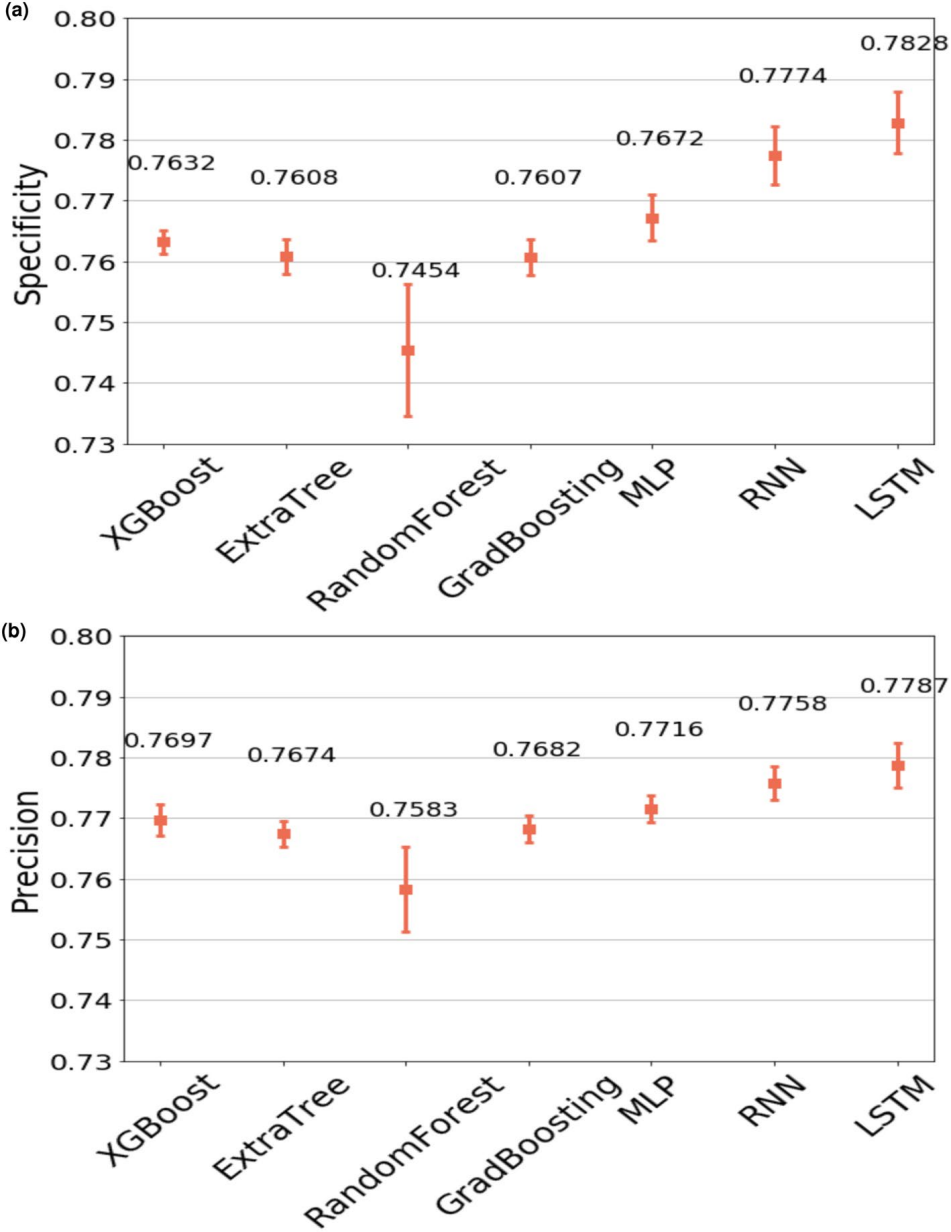
Comparisons of model performance. **(a)** The average of specificity values based on 5-fold cross validation. **(b)** The average of precision values of all models. The error bars represent the standard deviations to the mean values.

Table 1 presents detailed prediction performance and training time for all the models developed and assessed via 5-fold cross validation. All models achieved area under the receiver operating characteristic curve (AUC) scores higher than 0.8, which illustrates good performance in general for all the selected models in this classification task. The AUCs of XGBoost, Extra Trees, RF, GBM, MLP, RNN and LSTM are 0.8432, 0.8403, 0.8399, 0.8390, 0.8421, 0.8427 and 0.8426 respectively, Fig. 6 shows the mean AUCs for 7 selected models obtained from internal validation sets using cross validation, where the differences amongst AUCs were not statistically significant. However, it is worth noting that in the real world, our objective is to predict the patients who are less likely to adhere to their treatment plan so that further interventions can be introduced to this group before the scheduled medication day in an attempt to improve the adherence rate. In other words, the predictions of these who will be “Not On-Time” are more important than those who will be “On-Time”. Thus, the metric ‘specificity’, which describes the “True negative rate” is our focus in this study. Recognising this, from Table 1 we can see that, in general, the deep neural networks-based forecasting models outperformed the ensemble models. In particular, LSTM is the best performing model in this case and achieves the highest specificity value of 78.28% among all the models. Next, given the best performing model, LSTM, we conducted an ablation study to illustrate how different combinations of input features would have impact on the performance metrics of interest. The results of which are demonstrated in Table 2. Specifically, we carried out three set of representative experiments on the LSTM model. The first experiment only includes six historic drops as input features, which are essentially the dominant features constituting 94% of the feature importance as aforementioned in the “Feature analysis and selection”. The second experiment only includes the three most recent historic drops while the last experiment only contains the most recent drop. All six performance metrics are reported in the table, and likewise the ‘specificity’ of the model is still our main focus. It is clear that as we reduce the number of historic drops taken into account as input features, the specificity of the LSTM model degrades significantly from $76.92 \pm 0.58$ to $61.53 \pm 0.12$. Here, we note that with all six historical drops as the only input features, the LSTM model can achieve very promising results in comparison to the best result $78.28 \pm 0.51$ where all input features are used for training the LSTM model. In other words, this shows the minor importance of other feature combinations which is consistent with our findings in Fig. 5. Furthermore, Fig. 7 illustrates the two most significant performance metrics for this study, specificity and precision, where we can see the specificity of LSTM is 3.74% points higher than the RF model. In other words, LSTM has increased the ratio of the correct predictions made on the people who will not take medication on time (“Not On-Time” type) by 3.74% compared to RF, which has the lowest specificity. Similarly, Fig. 7b shows that the precision score of LSTM is also the highest of all and the value recorded is 2.04% points higher than RF.

**Table 1:** Comparisons of different models in terms of six performance metrics: AUC, Accuracy, Specificity, Sensitivity, Precision and F1 score.

Models	AUC	Accuracy (%)	Specificity (%)	Sensitivity (%)	Precision (%)	F1 score	Training time (s)
XGBoost	0.8432 ± 0.0008	77.73 ± 0.19	76.32 ± 0.34	79.15 ± 0.26	76.97 ± 0.25	0.7804 ± 0.0018	426.375
Extra Trees	0.8403 ± 0.0013	77.48 ± 0.19	76.08 ± 0.29	78.89 ± 0.25	76.74 ± 0.22	0.7780 ± 0.0019	38.352
RF	0.8399 ± 0.0011	77.20 ± 0.37	74.54 ± 1.09	79.85 ± 0.51	75.83 ± 0.70	0.7779 ± 0.0025	260.135
GBM	0.8421 ± 0.0009	77.70 ± 0.23	76.07 ± 0.29	79.33 ± 0.40	76.82 ± 0.22	0.7806 ± 0.0025	260.135
MLP	0.8421 ± 0.0005	77.67 ± 0.22	76.72 ± 0.37	78.62 ± 0.59	77.16 ± 0.22	0.7788 ± 0.0029	1072.451
RNN	0.8394 ± 0.0014	77.38 ± 0.20	77.74 ± 0.48	77.01 ± 0.63	77.58 ± 0.28	0.7729 ± 0.0027	528.575
LSTM	0.8390 ± 0.0013	77.35 ± 0.17	78.28 ± 0.51	76.42 ± 0.22	77.87 ± 0.36	0.7714 ± 0.0011	502.978

**Table 2:** Ablation study for the LSTM model in terms of six performance metrics: AUC, Accuracy, Specificity, Sensitivity, Precision and F1 score.

Input Features	AUC	Accuracy (%)	Specificity (%)	Sensitivity (%)	Precision (%)	F1 score
Six historic drops	0.8363 ± 0.0018	77.07 ± 0.21	76.92 ± 0.58	77.23 ± 0.44	76.99 ± 0.38	0.7711 ± 0.002
Three most recent historic drops	0.8168 ± 0.0026	76.47 ± 0.26	75.25 ± 0.46	77.69 ± 0.2	75.84 ± 0.34	0.7675 ± 0.0022
The most recent drop	0.7420 ± 0.0022	72.68 ± 0.18	61.53 ± 0.12	83.82 ± 0.28	68.54 ± 0.12	0.7542 ± 0.0018

**Figure 8:**
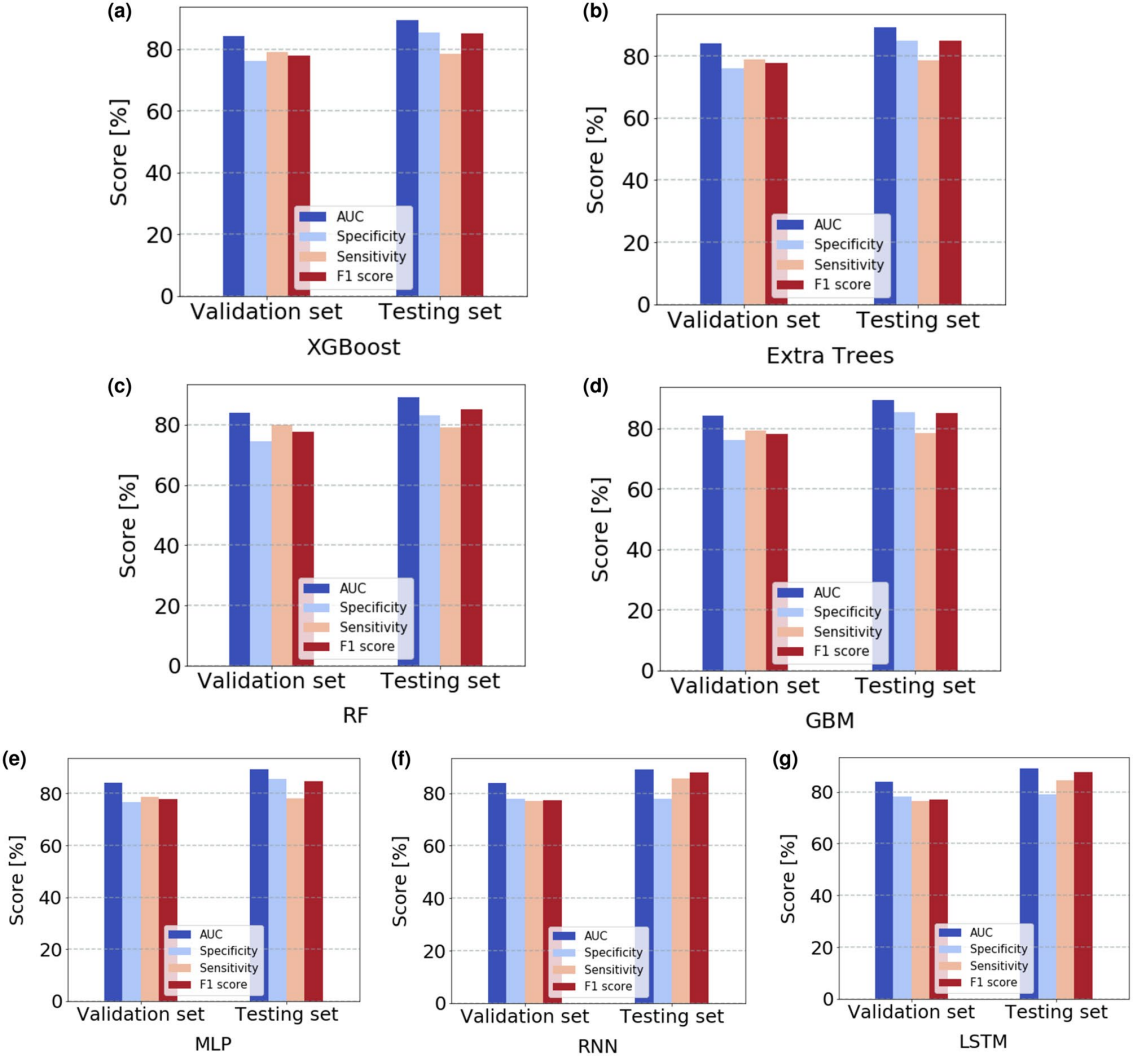
Model performance comparisons on validation and testing sets. The pre-trained models were deployed on future unseen testing set and achieved equivalent performance compared to that on the validation set.

As a more representative test of real world performance we deployed the pre-trained model based on an unseen future testing set and the predictions from the pre-trained model were compared with the ground truth of these patients’ real adherence behaviours. The performance of all models on this testing set is illustrated in Fig. 8. Four important performance metrics, AUC, specificity, sensitivity and F1 score were selected for comparison. We can see the performance of the models on this testing set is comparable or better than that in the internal validation set, which means the pre-trained models are not over-fitting and the proposed models generalize very well for the unseen dataset used in this case.

## Discussion

This study demonstrates the effectiveness of machine learning approaches in identifying patients who are at risk for future non-adherence within a specified time frame in the context of injected medications in a home setting. Based on the large-scale dataset collected from SSBs, the best performing machine learning model, LSTM, can predict the patients’ adherence to self-injections in a home setting with a high AUC of 0.8902 and an accuracy of 82.90% on the unseen testing set, which is much higher than a 50% random guess chance. The models we selected can be classified as ensemble learning and deep learning. Ensemble learning was found, not surprisingly, to be a robust and useful approach for this type problem, a fact well supported in the literature^12,39^. The good performance of ensemble learning is gained from the fusion of multiple heterogeneous classifiers that complement each other as a group. Bagging, one type of ensemble learning that combines multiple weak learners, learns independently from each other in a parallel manner, whereas, boosting connects classifiers sequentially in an adaptive way, where a newer model depends on the previous ones^40^. In this case, boosting can reduce the bias of the model and build a more complex and accurate model. This is consistent with our observations that among three ensemble learning models, we found that boosting approaches (XGBoost, GBM) outperformed bagging (RF, Extra Trees) although bagging is superior in terms of computational requirements for training. We also observed that both the RNN and LSTM yield better performance metrics (i.e. specificity) than a MLP approach, which is perhaps to be expected given that both RNN and LSTM models are more suitable for time series prediction. In terms of time efficiency to train the above models, the deep learning models typically required more time for training than bagging. On the other hand, boosting requires comparable training times to those required for the deep learning techniques. Hence, our experiments shows there is always a trade off between model performance and computational complexity. From Fig. 8, we can see the performance on testing set is comparable or even better than that on the internal validation set, an explanation of which, in terms of patient behavior, merits further investigation.

It should be noted that compared with previous studies which have predicted adherence based on data collected from multiple sources including patient-related features such as gender, age and race^41,42^, the approach here is primarily based on historic injection disposal records collected from the SSBs only. As a consequence we did not need to annotate any behavior with ground truth labels of adherence status thereby reducing sources of label noise. In addition the approach ensures that the data used is less prone to errors in collection, is consistent in format and collection methodology, and uniform in quality since opportunities for human error is greatly reduced. Another benefit of the approach described here is that the data is sourced from a wide range of patients, with different ethnicity, age, gender, even disease type. This suggests that the models developed and assessed have good generalisation properties despite variation in these factors across the dataset.

## Conclusion

In this work, a predictive analytics approach is used to demonstrate how event-based data can form the basis for a means for identifying patients who are at risk for future non-adherence. In the specific context of an IoT-enabled sharps bin (the SSB), the approach demonstrates how such data, produced in a home setting, can be harnessed to potentially create new approaches to benefit patients and health care providers. For example, there is a clear opportunity to use the prediction data to target patient interventions that can increase adherence for those patients who might benefit from such an approach. In tandem the approach reduces unnecessary intervention for those patients who can independently manage their own treatment. To appreciate the benefits of such a focused intervention, we can consider the scenario of a patient intervention system lacking such predictive analytics. Such a system may resort to the use of unconditional reminders to all patients, which can be conceptualised as a classifier that always predicts the minority class, i.e. that the patient will neglect to take their medication on time. However such unconditional notifications can lead to “notification fatigue” and alert desensitisation as there is no coupling between the intention state of the specific patient and the intervention system. Consequently, any adherence improvement may not be sustained. In contrast, with more accurate predictive analytics, reminders are rare events more closely coupled to a particular patient’s likely future behaviour and therefore come with increased likelihood of evoking attention and behavioural priming^43^. The broader impact of the data acquisition and predictive analytics described here concerns the validation of connected health approaches to the medical adherence problem and the intrinsic value of the data collected as the basis for ways to manage patients in such a context. As an example of this broader impact, it is worth noting that the IoT solution here that allows records of medication usage to be collected is clearly useful in terms of providing measures of adherence, it is the addition of machine learning which leads to the exploitation of structure within the data to produce models of future patient behaviour. This transformation from simple data records to models of anticipated patient behaviour enables a new conceptual shift in the development of connected health models. As a result the work presents further research opportunities to be addressed in the future. A particularly important aspect is the idea of developing trust in AI systems when used in the context established here. While the problem of trust in AI is complex, an important building block is explain-ability and the feature engineering approach adopted here gives some insight into how this might be obtained. For example, the large degree to which future behavior is predicted on relatively simple past behavior, i.e. recent history of adherence, lends itself very well to modern explainable approaches such as counterfactual explanations which rely on minimal attribute changes which flip the prediction label^44^. We would strongly encourage the exploration of such approaches for this problem to enhance trust and acceptance of AI in this important application area.

In conclusion, we have shown in this paper how the use of an IoT-enabled sharps bin integrating machine vision-based sensing can enable high quality assessment and capture of treatment events. We also validated and demonstrated that the data collected from the SSBs in combination with ensemble learning and deep learning models provides an accurate way of identifying patients who are at risk for future non-adherence. These valuable insights will enable targeted patient interventions. For example, it creates a patient-centred perspective that embraces a whole new set of possibilities to improve patient outcomes through interventions at the individual behavioural level through to re-structuring of care networks around specific patient groups, diseases and medication types.

## Data Availability

Authors Lara Kelly, Kieran Daly and Akshay Zalkikar are employees of Health Beacon Ltd, who are co-sponsors of the research. The data collected in this experiment used the Smart Sharps Bin developed by Health Beacon Ltd.

## References

[1] Sabaté, E. *et al.**Adherence to Long-Term Therapies: Evidence for Action* (World Health Organization, 2003).

[2] Cutler DM, Everett W (2010). Thinking outside the pillbox? Medication adherence as a priority for health care reform. N. Engl. J. Med..

[3] Miura T (2001). Effect of digoxin noncompliance on hospitalization and mortality in patients with heart failure in long-term therapy: A prospective cohort study. Eur. J. Clin. Pharmacol..

[4] Sellwood W, Tarrier N (1994). Demographic factors associated with extreme non-compliance in schizophrenia. Soc. Psychiatry Psychiatr. Epidemiol..

[5] Balkrishnan R, Christensen DB (2000). Inhaled corticosteroid nonadherence and immediate avoidable medical events in older adults with chronic pulmonary ailments. J. Asthma.

[6] Lecompte D, Pelc I (1996). A cognitive-behavioral program to improve compliance with medication in patients with schizophrenia. Int. J. Mental Health.

[7] Krueger KP, Berger BA, Felkey B (2005). Medication adherence and persistence: A comprehensive review. Adv. Ther..

[8] Cea-Calvo L (2020). Association between non-adherence behaviors, patients’ experience with healthcare and beliefs in medications: A survey of patients with different chronic conditions. Curr. Med. Res. Opin..

[9] Molfenter TD, Bhattacharya A, Gustafson DH (2012). The roles of past behavior and health beliefs in predicting medication adherence to a statin regimen. Patient Prefer. Adher..

[10] Cutrona SL (2012). Targeting cardiovascular medication adherence interventions. J. Am. Pharm. Assoc..

[11] Nelson A, Herron D, Rees G, Nachev P (2019). Predicting scheduled hospital attendance with artificial intelligence. Npj Digit. Med..

[12] Dietterich TG (2002). Ensemble learning. Handb. Brain Theory Neural Netw..

[13] Zhang, C. & Ma, Y. *Ensemble Machine Learning: Methods and Applications* (Springer, 2012).

[14] Sagi O, Rokach L (2018). Ensemble learning: A survey. Wiley Interdiscip. Rev..

[15] Karanasiou GS (2016). Predicting adherence of patients with HF through machine learning techniques. Healthc. Technol. Lett..

[16] Franklin JM (2016). Observing versus predicting: Initial patterns of filling predict long-term adherence more accurately than high-dimensional modeling techniques. Health Serv. Res..

[17] Nahmias DO, Civillico EF, Kontson KL (2020). Deep learning and feature based medication classifications from EEG in a large clinical data set. Sci. Rep..

[18] Chen J (2020). Deep learning-based model for detecting 2019 novel coronavirus pneumonia on high-resolution computed tomography. Sci. Rep..

[19] Yang Z, Bogdan P, Nazarian S (2021). An in silico deep learning approach to multi-epitope vaccine design: A Sars-Cov-2 case study. Sci. Rep..

[20] Pouyanfar S (2018). A survey on deep learning: Algorithms, techniques, and applications. ACM Comput. Surv..

[21] Liu W (2017). A survey of deep neural network architectures and their applications. Neurocomputing.

[22] Greff K, Srivastava RK, Koutník J, Steunebrink BR, Schmidhuber J (2016). Lstm: A search space odyssey. IEEE Trans. Neural Netw. Learn. Syst..

[23] Gu, Y., Zalkikar, A., Kelly, L., Daly, K. & Ward, T. E. Predicting injectable medication adherence via a smart sharps bin and machine learning. *arXiv preprint* arXiv:2004.01144 (2020).

[24] Ravì D (2016). Deep learning for health informatics. IEEE J. Biomed. Health Inform..

[25] Margffoy-Tuay, E. A., García-Hernandez, C. & Solano-Beltrán, D. C. Medication adherence improvement on rheumatoid arthritis patients based on past medical records. In *2018 IX International Seminar of Biomedical Engineering (SIB)* 1–6 (IEEE, 2018).

[26] Pettas, D., Nousias, S., Zacharaki, E. I. & Moustakas, K. Recognition of breathing activity and medication adherence using lstm neural networks. In *2019 IEEE 19th International Conference on Bioinformatics and Bioengineering (BIBE), IEEE* 941–946 (2019).

[27] Singh SM, Hanchate DB (2018). Improving disease prediction by machine learning. Int. J. Res. Eng. Technol..

[28] Mohebbi, A. *et al.* A deep learning approach to adherence detection for type 2 diabetics. In *2017 39th Annual International Conference of the IEEE Engineering in Medicine and Biology Society (EMBC)* 2896–2899 (IEEE, 2017). 10.1109/EMBC.2017.8037462 29060503

[29] Voigt, P. & Von dem Bussche, A. The EU general data protection regulation (gdpr). *A Practical Guide, 1st Ed., Cham: Springer International Publishing* (2017).

[30] Frank, E. *et al.* Weka-a machine learning workbench for data mining. In *Data mining and knowledge discovery handbook* 1269–1277 (Springer, 2009).

[31] Liu H, Zhou M, Liu Q (2019). An embedded feature selection method for imbalanced data classification. IEEE/CAA J. Autom. Sin..

[32] Maldonado S, López J (2018). Dealing with high-dimensional class-imbalanced datasets: Embedded feature selection for SVM classification. Appl. Soft Comput..

[33] Pedregosa F (2011). Scikit-learn: Machine learning in python. J. Mach. Learn. Res..

[34] Marchant, R. & Ramos, F. Bayesian optimisation for intelligent environmental monitoring. In *2012 IEEE/RSJ international conference on intelligent robots and systems* 2242–2249 (IEEE, 2012).

[35] Abadi, M. *et al.* Tensorflow: A system for large-scale machine learning. In *12th USENIX symposium on operating systems design and implementation (OSDI 16)* 265–283 (2016).

[36] Kingma, D. P. & Ba, J. Adam: A method for stochastic optimization. *arXiv preprint* arXiv:1412.6980 (2014).

[37] Liu, L. & Qi, H. Learning effective binary descriptors via cross entropy. In *2017 IEEE Winter Conference on Applications of Computer Vision (WACV)* 1251–1258 (IEEE, 2017).

[38] Hunter JD (2007). Matplotlib: A 2d graphics environment. Comput. Sci. Eng..

[39] Casanova R (2014). Application of random forests methods to diabetic retinopathy classification analyses. PLoS ONE.

[40] Freund Y, Schapire R, Abe N (1999). A short introduction to boosting. Jpn. Soc. Artif. Intell..

[41] Chan DC (2010). Patient, physician, and payment predictors of statin adherence. Med. Care.

[42] Theofilou P (2013). The effect of sociodemographic features and beliefs about medicines on adherence to chronic kidney disease treatment. Int. J. Caring Sci..

[43] Doyen S, Klein O, Pichon C-L, Cleeremans A (2012). Behavioral priming: It’s all in the mind, but whose mind?. PLoS ONE.

[44] Keane, M. T. & Smyth, B. Good counterfactuals and where to find them: A case-based technique for generating counterfactuals for explainable ai (xai). In *International Conference on Case-Based Reasoning* 163–178 (Springer, 2020).

